# Genome Sequences of Marine Shrimp *Exopalaemon carinicauda* Holthuis Provide Insights into Genome Size Evolution of Caridea

**DOI:** 10.3390/md15070213

**Published:** 2017-07-05

**Authors:** Jianbo Yuan, Yi Gao, Xiaojun Zhang, Jiankai Wei, Chengzhang Liu, Fuhua Li, Jianhai Xiang

**Affiliations:** 1 Key Laboratory of Experimental Marine Biology, Institute of Oceanology, Chinese Academy of Sciences, 7, Nanhai Road, Qingdao 266071, China; yuanjb@qdio.ac.cn (J.Y.); gyocean@qdio.ac.cn (Y.G.); liu@qdio.ac.cn (C.L.); fhli@qdio.ac.cn (F.L.); 2 Laboratory for Marine Biology and Biotechnology, Qingdao National Laboratory for Marine Science and Technology, 1, Wenhai Road, Qingdao 266071, China; 3 Ocean University of China, 5, Yushan Road, Qingdao 266071, China; weijiankai@ouc.edu.cn

**Keywords:** *Exopalaemon carinicauda* Holthuis, genome, caridean shrimp, genome size evolution

## Abstract

Crustacea, particularly Decapoda, contains many economically important species, such as shrimps and crabs. Crustaceans exhibit enormous (nearly 500-fold) variability in genome size. However, limited genome resources are available for investigating these species. *Exopalaemon* *carinicauda* Holthuis, an economical caridean shrimp, is a potential ideal experimental animal for research on crustaceans. In this study, we performed low-coverage sequencing and *de novo* assembly of the *E. carinicauda* genome. The assembly covers more than 95% of coding regions. *E. carinicauda* possesses a large complex genome (5.73 Gb), with size twice higher than those of many decapod shrimps. As such, comparative genomic analyses were implied to investigate factors affecting genome size evolution of decapods. However, clues associated with genome duplication were not identified, and few horizontally transferred sequences were detected. Ultimately, the burst of transposable elements, especially retrotransposons, was determined as the major factor influencing genome expansion. A total of 2 Gb repeats were identified, and RTE-BovB, Jockey, Gypsy, and DIRS were the four major retrotransposons that significantly expanded. Both recent (Jockey and Gypsy) and ancestral (DIRS) originated retrotransposons responsible for the genome evolution. The *E. carinicauda* genome also exhibited potential for the genomic and experimental research of shrimps.

## 1. Introduction

Crustacea (such as shrimps and crabs) and Hexapoda (mainly insects) are two species-rich groups of Arthropoda, which is the phylum with the highest number of species identified worldwide [1]. Abundant genome resources have been identified from insects (more than 40 species), whereas few crustaceans genomes, except those of the branchiopod *Daphnia pulex* and the amphipod *Parhyale hawaiensis* [2,3], have been completely sequenced. In particular, the genomes of decapods are difficult to sequence and assemble due to the large size and complexity of the genome; these economically important species include *Litopenaeus vannamei*, *Penaeus monodon*, *Macrobrachium rosenbergii*, *Exopalaemon carinicauda*, and *Eriocheir sinensis* [4,5]. Nevertheless, the low-coverage sequencing of the cherry shrimp *Neocaridina denticulata* and the Chinese mitten crab *E. sinensis* impels research on the genomic characteristics of crustaceans [6,7].

*E. carinicauda* Holthuis, which belongs to the family Palaemonidae of Crustacea, is an economically important shrimp species in China; the production of this species ranks only third to that of *Fenneropenaeus chinensis* and *Acetes chinensis* in the coast of China [8]. In addition to its economic value, *E. carinicauda* is a potential ideal experimental animal for research on crustaceans because of its moderate size, transparent body, strong tolerance to environmental stress, big egg (egg diameter ranges from 0.57 mm to 1.08 mm), and high reproduction capacity [5,9]. *E. carinicauda* can survive at temperature of 2 °C–38 °C, salinity of 4–35‰, and pH of 4.8–10.5, and thus can be easily cultured in laboratory [5]. Moreover, this species possesses a high reproductive capacity at a reproduction cycle of only 2 months all year round [9]. Therefore, *E. carinicauda* has been widely used as an experimental animal for identifying functional genes in crustaceans [10,11]. Moreover, genome editing approach was first successfully applied to the embryos of *E. carinicauda* by using CRISPR/Cas9 system in our group recently [12]; as such, this species could be a novel model organism for research on decapod shrimps to reveal the function of genes relevant to growth, development, and reproduction.

Genome size varied considerably among crustaceans that ranged from 0.14 pg (*Cyclops kolensis*) to 64.62 pg (*Ampelisca macrocephala*), resulting in nearly 500-fold variation [13]. According to the Animal Genome Size Database (www.genomesize.com), the *C*-values of shrimps from Palaemonidae range from 6.48 pg to 22.16 pg, implying about 3.4-fold variation in the genome size, which was similar to that of snapping shrimps [14]. In this regard, factors affecting genome size evolution were important evolutionary biology issue. Generally, whole-genome duplication and proliferation of large-scale transposable elements mainly influence genome expansion, which is even associated with species divergence [14,15,16,17]. Besides, horizontal gene transfer (HGT) also contributes to genome evolution, and more frequently occurs in prokaryotes than in eukaryotes [18,19]. The largest genomes and range of crustaceans are found within the class Malacostraca, with many caridean shrimps harboring relative larger genomes [20,21]. *E. carinicauda* is a representative species of caridean shrimps for research on genome size evolution.

Despite that *E. carinicauda* could be a potential experimental animal for crustacean research, the genome resources are limited, especially to genome editing experiments. Thus, in this study, we performed low-coverage sequencing of *E. carinicauda* and obtained draft genome sequences for investigating genomic characteristics, including heterozygosity, repeat sequences, functional gene clusters, and phylogenetical position. We also identified factors affecting genome size evolution of caridean shrimps.

## 2. Results

### 2.1. Genomic Characteristics

**Figure 1:**
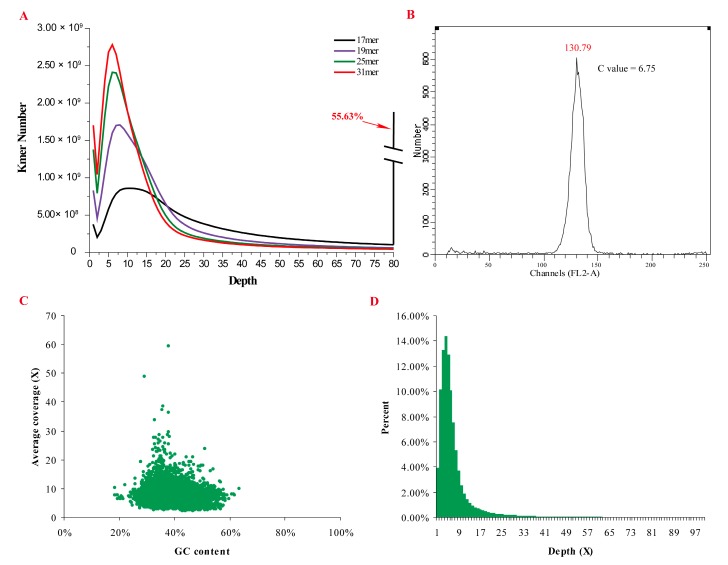
Genome characteristics of *E. carinicauda*. (**A**) K-mer distribution of the sequencing data with the K-mer size of 17, 19, 25, and 31. (**B**) Flow cytometry results of shrimp appendages. (**C**) Plot of GC content against the average sequencing depths of contigs longer than 1.5 Kb. The scatter points clustered together indicated none heterozygous sequences had been found in the assembled genome. (**D**) Sequencing depth distribution of the bases throughout the genome.

A total of 88 Gb high quality Illumina clean data were obtained for genome assembly (Supplementary Table S1, Figure S1). To assess the effect of heterozygosity and repeats on the assembly, we performed K-mer analysis of the clean reads. In contrast to the bimodal distribution of K-mer frequencies in *L. vannamei* and *P. hawaiensis* [3,4], a single peak was observed in *E. carinicauda*; this peak corresponds to K-mers present on homozygous regions (Figure 1A). No obvious low coverage peak was observed in *E. carinicauda* compared with the highly heterozygous genome of *Crassostrea gigas* and *P. hawaiensis* [3,22], indicating low levels of heterozygosity. Moreover, a high proportion (55.63%) of K-mers with depth higher than 80× indicated the presence of abundant repetitive sequences involved in the *E. carinicauda* genome.

We performed flow cytometry analysis on appendage cells to assess genome size of *E. carinicauda*. The haploid genome size was estimated as 6.62 Gb with C-value of 6.75 pg (1 pg = 978 Mb) (Figure 1B). Based on the K-mer analysis, the genome size was estimated to be 5.73 Gb, which is relatively lower than the flow cytometric results. The genome size of *E. carinicauda* is higher than those of *D. pulex* (197 Mb) [2], penaeid shrimps [4,23], and another caridean shrimp *N. denticulata* (3 Gb) by 29-, 2.5- and 2-folds, respectively [24]. Although smaller than some decapods (e.g., krill has genome size of ~47 Gb) [25], *E. carinicauda* has relatively larger genome size than many commonly recognized decapod shrimps.

To understand polymorphism in the *E. carinicauda* genome, we analyzed allelic variation in the assembled genome. A total number of 8,918,607 single-nucleotide polymorphisms (SNPs) and 3,342,259 short insertion/deletion (Indels) were obtained from the genome, yielding a sequence polymorphism rate of 0.18%, which is lower than that of highly polymorphic genomes [3,22].

### 2.2. Genome Assembly and Validation

*De novo* assembly of the *E. carinicauda* genome was conducted using SOAPdenovo software. A total of 20,407,032 contigs with N50 length of 263 bp and 13,897,062 scaffolds with N50 length of 816 bp were produced (Table 1). The total length of the assembled scaffolds was 5.57 Gb, which covered approximately 97.17% of the genome. As shown in Figure 1C, all the contigs accumulated without low-coverage sequences. Additionally, a single peak was detected on the sequencing coverage distribution plot (Figure 1D), indicating that few heterozygous sequences were involved in the genome.

Over 96% of the Illumina sequencing reads were successfully mapped to the genome, reflecting the high integrity and accuracy of the assembly. Of the 2675 conserved core genes of Arthropoda used to assess genome completeness, 1767 genes (66.06%) were covered by the genome as estimated by BUSCO program, which was similar to that of *P. hawaiensis* (65.49%) [3]. As many core genes may be lost during the divergence of crustaceans, we remapped these core genes to the genome after excluding the core genes both missed in *E. carinicauda* and *P. hawaiensis*. Thus, about 88.44% core genes were covered by *E. carinicauda* genome, and among them, there are 868 (43.44%) complete and single-copy BUSCOs. To further examine the completeness of the assembly, we collected the transcriptome data of *E. carinicauda* and aligned the assembled unigenes against the genome. A total of 81,135 unigenes with mean length of 1064 bp were obtained (Supplementary Table S2). Over 95% of the unigenes were mapped to the genome, and more than 83.96% of the unigenes were covered by single scaffold in half length (Table 2). Therefore, the assembled genome displayed high completeness, especially for coding regions.

### 2.3. Phylogenetic Location

The unigenes of 10 decapods were produced from the transcriptome data, and the quantity of unigenes ranged from 66,815 (*L. vannamei*) to 133,311 (*A. leptodactylus*). A total of 19,630 gene families were collected based on the comparative analysis of protein-coding genes of the 12 crustaceans. Among these gene families, a subset of 202 single-copy gene families was selected for phylogenetic analysis. Sequences from the 202 genes, totaling 46,653 amino acids, were applied for phylogenetic tree construction using Maximum likelihood (ML) and Bayesian inference (BI) algorithms. The topology of the ML tree was similar to that of BI tree, and the support values were almost 100% on each branch.

**Figure 2:**
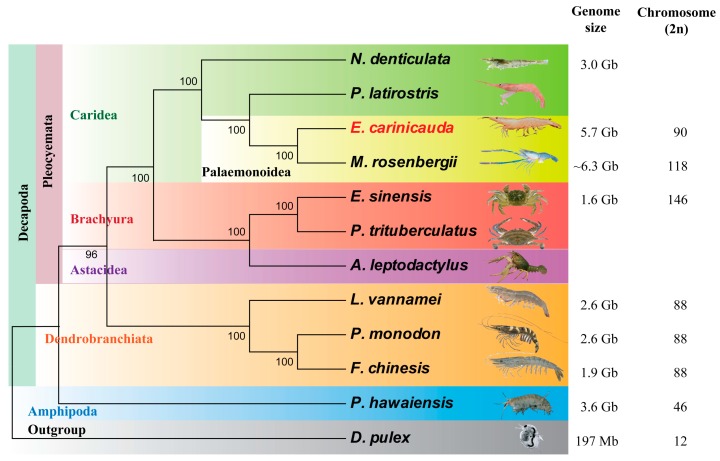
Phylogenetic tree of decapod species. ML tree obtained with a super-matrix of 46,653 amino-acid residues gathered from 202 orthologous genes in 10 decapod shrimps and two outgroup species, *P. hawaiensis* and *D. pulex*. The genome size and chromosome numbers (2*n*) of other species were obtained from previous researches [2,3,4,6,7,21,23,30,31]. The genome size of *M. rosenbergii* was replaced by *C*-value of *Macrobrachium acanthurus* from the Animal Genome Size Database.

On the phylogenetic tree (Figure 2), caridean (Caridea) and penaeid shrimps (*Dendrobranchiata*) were both monophyletic. Caridea, Brachyura, and Astacidea shared a common ancestor of Pleocyemata, consistent with many previous reports [7,26,27], but different from those constructed using 16S rRNA, 18S rRNA, 28S rRNA and H3 genes [28,29], which grouped Caridea and Penaeoidea together. As expected, *M. rosenbergii* was phylogenetically close to *E. carinicauda* that supported Palaemonidae.

### 2.4. Assessment of Genome Duplication

*E. carinicauda* possesses relatively larger genome size than many other crustaceans. In this regard, we examined factors affecting genome expansion; these factors include genome duplication events, HGT events, and TE expansion. *E. carinicauda* contains 45 pairs (2*n* = 90) of chromosomes (Figure 2), which is similar to that of penaeid shrimps (2*n* = 88) [4,23,30], but less than that of *M. rosenbergii* (2*n* = 118) [31]. However, the genome size of *E. carinicauda* is larger than those of penaeid shrimps and the caridean shrimp *N. denticulata*, but close to that of *M. rosenbergii*. Therefore, this evidence suggests the *E. carinicauda* genome is unlikely to be polyploid.

**Figure 3:**
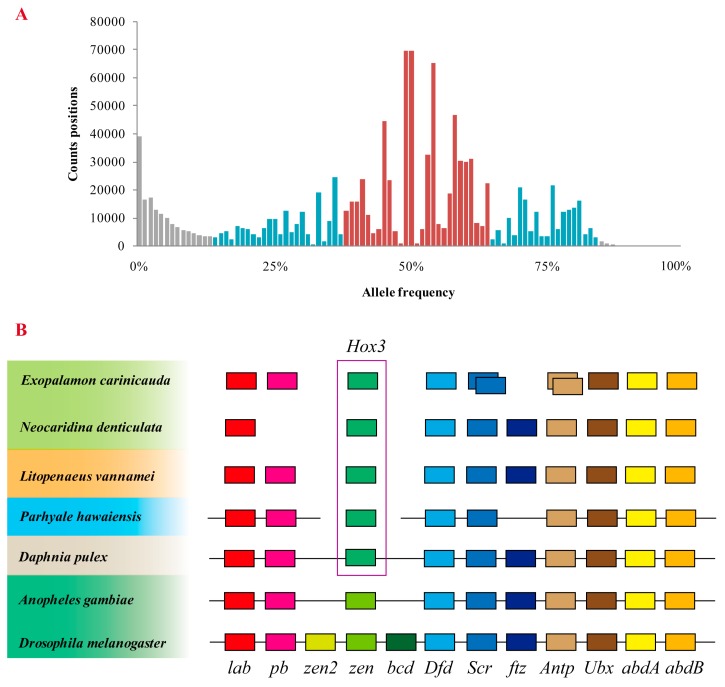
Allele frequency spectra and Hox gene cluster. (**A**) Allele frequency spectra based on read counts of bi-allelic SNPs. (**B**) Hox gene cluster in seven arthropods. The box linked with a straight line indicates the ordered genes located on a single scaffold or linkage groups.

Alleles frequency was calculated to identify genome duplication events. A total of 1,196,796 bi-allelic SNPs were used for the analysis. As expected, a unimodal distribution was detected (Figure 3A), with a peak at 50%, which support *E. carinicauda* is a diploid. No obvious peaks at 25% and 75% was found, indicating genome duplication may have not happened in the *E. carinicauda* genome.

Furthermore, we investigated the copy number of the Hox gene cluster and single-copy genes. We identified nine of the 10 Hox genes (except *ftz*) in the *E. carinicauda* genome (Figure 3B), similar to that of *P. hawaiensis* [3]. Seven of the nine genes have single copies in the genome, except for *Scr* and *Antp*, which possessed two copies. Analysis of single-copy genes is another effective method used to evaluate genome duplication, because they only have one physical location in the genome and single orthologs in other species. Of the 202 single-copy genes collected from comparative transcriptomic analysis, 176 genes (87.13%) have only one copy in the genome. The 26 remaining genes displayed partial duplication (cover more than 10% of unigene) on different scaffolds. Analysis of the Hox gene cluster and single-copy genes indicated that these genes mostly had single copy in the genome, although few of them duplicated. Therefore, it was reasonable to believe that no genome duplication events occurred during the evolution of the *E. carinicauda* genome.

### 2.5. Horizontally Transferred Sequences

**Figure 4:**
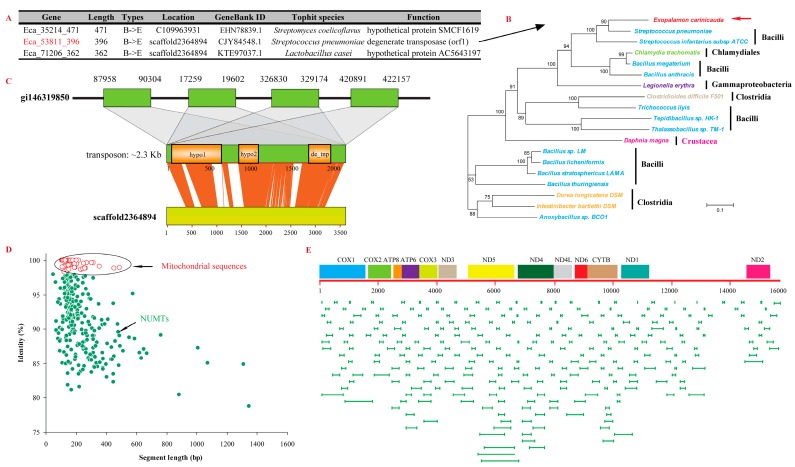
Horizontally transferred sequences of the genome. (**A**) Three bacteria-originated candidate HTGs of *E. carinicauda*. (**B**) Phylogenetic tree of the candidate HTG (Eca_53811_396). (**C**) Structures of probable horizontally transferred DNA fragments and their locations in both the donor and receptor genome. The syteny between shrimp genomic contigs and corresponding donor genomes are displayed. de_tnp indicates the gene encode degenerate transposase. hypo1 and hypo2 are two genes encode hypothetical proteins. (**D**) The identity distribution of mitochondrial genome against the genome. The red circle with high identity values are mitochondrial sequences from the assembled genome, while the green circle with relative low identity values are NUMTs. (**E**) The location of the NUMTs along the mitochondrial genome.

Horizontally acquired mobile elements, such as bacteriophages, mitochondria, and transposable elements, can contribute to genome plasticity, resulting in divergence in genetic materials [32,33]. In the present study, we used an exhaustive detection method to identify horizontally transferred genes (HTGs) in *E. carinicauda*. When the 81,135 unigenes were blasted against the prokaryote genome database, 562 homologs were collected; of which, 16 candidate HTGs that showed homologous to the genome were used for subsequent phylogenetic analysis. Finally, three candidate HTGs, all bacteria originated, were identified in the *E. carinicauda* genome (Figure 4A). For these candidate HTGs, the phylogenetic tree showed a topology of *E. carinicauda* nesting with bacteria but far from other eukaryotes (*Daphnia magna*), indicating a bacteria-to-*E. carinicauda* HGT event (Figure 4B). Two of the three candidate HTGs encoded hypothetical proteins, and the other one (*de_tnp*) encoded degenerate transposase, which is involved in a degenerate transposon in the most probable donor genome, *Streptococcus pneumoniae* [34]. When comparing the genome of *S. pneumoniae* against the *E. carinicauda* genome, four copies of transposon (about 2.3 Kb) around *de_tnp* were found in *S. pneumoniae*, and were found to be homologous to the scaffold2364894 (Figure 4C). However, these sequences could also be contaminating sequences, that need further confirmation. In comparison with *E. carinicauda*, 21 candidate HTGs were identified in *N. denticulata* after homology detection of unigenes against prokaryote genome database, arthropod protein database, *N. denticulata* genome and NCBI non-redundant protein (Nr) database. Additionally, the horizontally transferred degenerate transposon in *S. pneumoniae* was also detected in the *N. denticulata* genome, indicating it may be horizontally transferred before the divergence of these two shrimps.

Horizontal transfer events also frequently occur between mitochondrial genome and nuclear genome, thereby generating large amounts of nuclear mitochondrial DNA segments (NUMTs) [35]. The complete mitochondrial genome of *E. carinicauda* was covered by 12 scaffolds, with homology identity larger than 98% (Figure 4D). A total of 177 NUMTs (total length of 67,764 bp) with relatively low homology identity (≤98%) were inferred in the genome; this number is significantly higher than that of *N. denticulata* (35 NUMTs has total length of 4718 bp) and *D. pulex* (1 NUMTs with length of 3800 bp) (*p* ≤ 0.01). The longest NUMTs in *E. carinicauda* was 8054 bp, and the mean length of NUMTs was 385 bp, which was also longer than *N. denticulata* (longest: 268 bp, mean length: 127 bp). The mitochondrial genome coverage by NUMTs was significantly higher in *E. carinicauda* (92.88%) than that in *N. denticulata* (26.25%) and *D. pulex* (24.21%) (*p* ≤ 0.01). The mitochondrial genome of *E. carinicauda* was almost equally covered by NUMTs several times, except the region of 13.3–14.3 Kb, which with nearly none of NUMT covered and none of the genes located (Figure 4E). Basing on the transcriptome data, we found that seven genes (including two genes encoding NADH2, and genes encoding NADH5, COI, COIII, and cytob) on NUMTs could be transcribed, which implied that some of these HTGs might be functional in the nuclear genome.

A large amount of NUMTs were horizontally transferred from the mitochondrial genome to the nuclear genome in *E. carinicauda*. To identify whether the burst of NUMTs originates from duplication event after the transfer [36], we compared the homology identity distribution of the mitochondrial genome-NUMTs to NUMTs-NUMTs (Supplementary Figure S2). It was found that a bit more NUMTs showed higher identity (95–98%) to the mitochondrial genome than other NUMTs, indicating that these NUMTs were more likely originated from the mitochondrial genome rather than by NUMT duplication.

### 2.6. TE Expansion

TE expansion is a major factor that causes genome expansion in many species. Through using RepeatModeler and RepeatMasker, we annotated the repetitive sequences of the *E. carinicauda* genome and compared them with those of *N. denticulata*, *P. hawaiensis*, and *D. pulex*. A total of 2 Gb (36.37% of genome) repeats were annotated in the *E. carinicauda* genome (Supplementary Table S3); and among them, simple repeats accounted for 1.39% of the genome. Long interspersed nuclear elements (LINEs, 8.86%) and long terminal repeats (LTR, 5.41%) were found to be the two major classes of transposable elements (TEs) that accounted for the *E. carinicauda* genome. Moreover, RTE-BovB (3.36%), Jockey (2.12%), and CR1 (1.65%) were the three major components of LINEs. Gypsy (2.41%) and DIRS (2.87%) were the two major classes of TEs under LTR. When compared the four crustaceans, SINEs were commonly fewer than other TEs, and the composition of other TEs significantly differed among the four species (Table 3). *E. carinicauda* had relatively higher LINEs than other three crustaceans, especially for RTE-BovB and Jockey (*p* ≤ 0.01). Besides, *E. carinicauda* had relatively more LTRs than *N. denticulata* and *P. hawaiensis*, especially for Gypsy and DIRS, which were significantly more than the two species (*p* ≤ 0.01). Therefore, RTE-BovB, Jockey, Gypsy, and DIRS were four retrotransposons (totally 474 Mb) that significantly expanded in the *E. carinicauda* genome.

**Figure 5:**
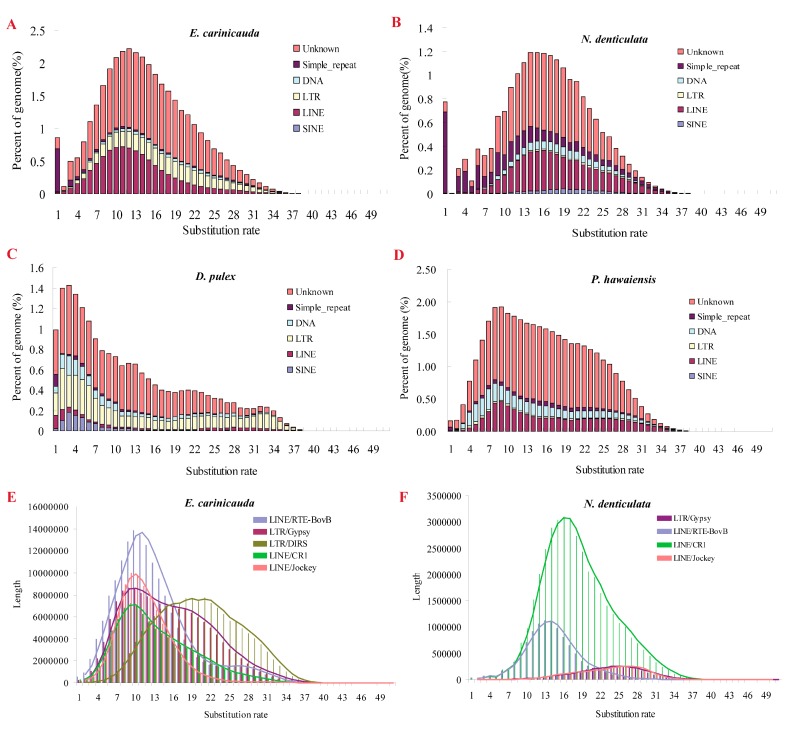
Divergence distribution of the repeats compared to consensus in the TE library. The divergence time of repeats were compared among four species: (**A**) *E. carinicauda*, (**B**) *N. denticulata*, (**C**) *D. pulex*, (**D**) *P. hawaiensis*. The divergence time of expanded LINEs and LTRs were compared between (**E**) *E. carinicauda* and (**F**) *N. denticulata*.

The burst of TEs greatly contributed to the genome expansion of *E. carinicauda*. To study the divergence history of TEs in the genome, we calculated the divergence rate between the identified TEs and the consensus sequences in the TE library. A single peak around 12% was detected in the substitution rate distribution of TEs in the *E. carinicauda* genome (Figure 5A), indicating that the TEs expanded at the same time. Similar results were detected in the genome of another caridean shrimp *N. denticulata* (Figure 5B). The substitution rate distribution of *P. hawaiensis* and *D. pulex* showed minimal differences in the peaks at higher rates (rates ≥ 20%), indicating the ancient expansion of TEs (Figure 5C,D). Many TEs were no longer active, and various TEs might have different amounts of active members because of different divergence histories [37]. Therefore, we investigated the divergence histories of the five expanded retrotransposons (RTE-BovB, Jockey, CR1, Gypsy, and DIRS) of *E. carinicauda* (Figure 5E). The substitution rate for DIRS (21%) was twice higher than that for three LINEs (RTE-BovB Jockey, and CR1, 10%); hence, DIRS were older than RTE-BovB, Jockey, and CR1, which might retain relatively more active copies. Besides, two peaks were found on the substitution rate distribution of Gypsy, accounting for ancient and recent expansion. Similar result was observed in *N. denticulata* (Figure 5F), but only ancient expansion was detected in Jockey and Gypsy.

## 3. Discussion

### 3.1. E. carinicauda Possesses a Large Complex Genome

In this study, we performed low-coverage sequencing on the *E. carinicauda* genome and investigated its characteristics. Genome survey analysis indicates that *E. carinicauda* has a relatively large genome size of 5.73 Gb, similar to many other caridean shrimps as shown in the Animal Genome Size Database. The genome size of *E. carinicauda* is 29-fold higher than that of *D. pulex*, and more than twice of that of penaeid shrimps. Despite originating from the same infraorder Caridea, *E. carinicauda* and *M. rosenbergii* have genome size twice higher than that of *N. denticulata* (Figure 2). Caridean shrimps harbor large genomes within the class Malacostraca, which have the largest genomes and range among crustaceans [20,21]. Therefore, genome survey of *E. carinicauda* provides a valuable resource for investigation of the features of large caridean genomes.

K-mer analysis indicated that *E. carinicauda* has relatively low level of heterozygosity and high frequency of repetitive sequences. We annotated about 2 Gb repeats, which account for 36.37% of the *E. carinicauda* genome. Some of structural repeats could not be annotated because of the highly fragmentation of the genome assembly, resulting in underestimation of the repeats in the genome. Therefore, *E. carinicauda* has a genome with large amount of repeats. Moreover, *E. carinicauda* has more than 76 Mb (1.39%) simple repeats, which is higher than that of many other invertebrates, such as *C. gigas* (0.72%), *D. pulex* (0.44%), and *Lottia gigantea* (0.55%). Even harboring similar genome size, the locust *Locusta migratoria* (6.5 Gb) has only 0.2% of simple repeats. Therefore, large amounts of repeats, including simple repeats, complicate the genome of *E. carinicauda*.

*E. carinicauda* possesses a large complex genome; as such, we made efforts to recover the full genome and assembled a genome of 5.57 Gb. This genome effectively covered the protein-coding regions, showing that the coverage of core genes and 81,135 unigenes exceeds 88% and 95%, respectively. Furthermore, we found 40,002 unigenes, which could be almost fully covered by single scaffold (Table 2), providing a valuable resource to obtain complete genes. Hence, deep genome sequencing, including PacBio long reads sequencing, will promote genome investigation of *E. carinicauda*.

### 3.2. Burst of TEs Responsible for Genome Expansion of E. carinicauda

Genome size varies considerably among species; and genome duplication, HGT events and TE expansion are considered to be potential factors causing genome expansion [14,15,16,17,18,19]. Whole-genome duplication can lead to rapid genome expansion, followed by large-scale chromosomal rearrangements and deletions during polyploidization [38]. Whole-genome duplications are commonly detected in plants and some animals (e.g., fishes), but rarely in crustaceans (except Branchiopoda) [39,40]. The Atlantic horseshoe crab *Limulus polyphemus* (Arthropoda, Chelicerata) and many other horseshoe crabs, which is phylogenetically close to Crustacea, underwent ancient whole-genome duplication of two clusters of Hox genes in different linkage groups [41,42]. Besides, gene duplications can also imply to the protein domain duplication that doubles the transcript size [43]. However, only one cluster of Hox genes was identified in the genome of *E. carinicauda*, and most orthologous single-copy genes (87.13%) remained as single copy on the genome. Furthermore, the average coverage of sequencing reads on the contigs with Hox genes and single-copy genes showed a single peak around 7×, similar to that of full contigs (Supplementary Figure S3). Therefore, the Hox gene cluster and single-copy genes were possibly not duplicated, suggesting that *E. carinicauda* did not undergo genome duplication. This finding is also supported by the research on snapping shrimps, which were not polyploid because their genome size was not related to chromosome number [14].

In general, the effects of HGT events on genome plasticity are mainly discovered in prokaryotes [18,19]. In this study, only three candidate HTGs were identified in the *E. carinicauda* genome. Moreover, only 16 candidate unigenes of *E. carinicauda* showed homology to prokaryotic genomes, and only 21 candidate HTGs were identified in *N. denticulata*. Compared with *N. denticulata* (35 NUMTs with total length of 4718 bp), a relatively larger amount of NUMTs (177 NUMTs with total length of 67,764 bp) were detected to be horizontally transferred to the nuclear genome of *E. carinicauda*. Overall, all these horizontally transferred sequences minimally influenced genome size evolution.

For many species, TEs are generally the major components of complex genomes and their transposition can be regarded as predominant force driving genome expansion [16,17,44,45,46,47]. Besides, retrotransposons are considered a particular class of TEs that greatly contribute to genomic inflation because of their propensity to increase the copy numbers [16,48]. *E. carinicauda* contains approximately 2 Gb repeats, and LINEs and LTRs were found to be the two major retrotransposons that significantly proliferated in the genome. Among them, RTE-BovB, Jockey, Gypsy, and DIRS significantly expanded compared with those of the other three crustaceans. It seems the four kinds of retrotransposons are specifically responsible for the genome size evolution of *E. carinicauda*. Furthermore, these retrotransposons appeared to be relatively recent transposed after the divergence of *E. carinicauda* or its ancestor. SINEs was considered as the major TEs that contribute to species-specific genome sequences [49]. However, few SINEs were found in the four genome-sequenced crustaceans (Table 3), suggesting that these species-specific repeats minimally contributed to remodeling the genomes of *E. carinicauda* or other crustaceans.

Many retrotransposons are common and presumably of ancestral origin, so many of their members are no longer active [37]. Therefore, recently transferred TEs are more active and exert more contribution to remodel genome. In both caridean shrimps, *E. carinicauda* and *N. denticulata* have a single peak of substitution rate distribution of TEs (Figure 5), suggesting that the burst of TEs occurred at the same time. In contrast to that of *D. pulex* and *P. hawaiensis*, which seemed to have ancestral repeats, the two caridean shrimps seemed to have only recent repeats. Since both caridean shrimps share similar genomic structures, we speculated about the cause of differences in genome size. When comparing these two shrimp species, RTE-BovB, Jockey, Gypsy, and DIRS significantly expanded in the *E. carinicauda* genome (Table 3). The divergence time of RTE-BovB and CR1 were similar in the two shrimp species that occurred recently, whereas the divergence time of Jockey, Gypsy, and DIRS were different from one another. A recent burst of Jockey and Gypsy, and a relative ancestral origin of DIRS was found in the *E. carinicauda* genome, indicating that both recent and ancestral origin of retrotransposons contributed to genome expansion, but different classes of retrotransposons expanded at different times.

Comparison of substitution rates for DIRS and some other TEs (e.g., RTE-BovB, Jockey, and Gypsy) indicated that DIRS is a relatively old repeat family. Unlike most other retrotransposons, DIRS encodes a tyrosine recombinase that is involved in site-specific recombination. DIRS has been identified in a wide range of eukaryotes, including fungi, plants and various animals, but, few of them have been reported in arthropods [50]. In *N. denticulata* and *P. hawaiensis*, no DIRS were identified, but about 143 Mb (2.87%) DIRS were detected in *E. carinicauda*. Therefore, we hypothesize that DIRS may stem from horizontal transfer from related species. To test this hypothesis will require full-length annotated TE sequences and additional comparative sequences. Jockey and Gypsy were younger than DIRS that they may have many likely active copies, which would contribute to genome evolution. Jockey and Gypsy are two kinds of retrotransposons widely identified in animals and plants. However, Gypsy showed some differences from Jockey, indicating that a part of Gypsy expanded at a time similar to that of DIRS (Figure 5E). Therefore, unlike other retrotransposons, Gypsy underwent two times of expansion during the genome evolution.

## 4. Materials and Methods

### 4.1. Sample Preparation and Sequencing

Sample for genome sequencing was collected from an adult male *E. carinicauda* cultured in the aquaculture lab of IOCAS, Qingdao, China. The animal was starved and acclimated in sea water aquaria at 20 ± 1 °C for one week. Muscles were collected immediately, frozen in liquid nitrogen, and stored at −80°C. Genomic DNA was isolated from the muscles by using a TIANamp Marine Animal DNA Kit (TIANGEN, Beijing, China) according to the manufacturer’s instructions. Short-insert (170 bp and 500 bp) DNA libraries were constructed according to Illumina manufacturer’s protocol. All libraries were sequenced on the Illumina sequencing platform HiSeq2000 with the paired-end (PE) sequencing reads length of 100 nt. All the sequencing reads were trimmed to filter the adaptor sequences and low-quality reads by using NGS QC Toolkit [51]. ErrorCorrection v2.0.1 from Short Oligonucleotide Analysis Package (SOAP) (http://soap.genomics.org.cn/index.html) was used to correct read error with default parameters. All the sequencing data has been deposited on GeneBank SRA database with the accession numbers of SRR5320375 and SRR5320376.

### 4.2. Estimation of Genome Size, Heterozygosity, and Repetitiveness

Genome size was determined using flow cytometry. Appendages were collected from 10 *E. carinicauda* individuals (for 10 replicates), and mouse (genome size of 2.50 Gb) blood cells were used as internal standard. Briefly, samples of tissues were chopped with a razor blade in the buffer of PBS. 1 mL of the homogenized cell suspension was filtered through a 30 µm nylon filter, added with 12 µL of propidium iodide (50 mg/mL), and stained with 2 µL of RNase (10 mg/mL) for 20 min. All samples were run on a flow cytometer BD FACSCalibur (BD Biosciences, San Jose, CA, USA) by using a 488 nm blue laser to obtain single-parameter histograms showing relative fluorescence between the standard nuclei and shrimp nuclei.

Additionally, genome size was estimated based on the K-mer (K represents the chosen length of substrings) depth distribution of shotgun reads; this method has been used to accurately estimate the genome size of a number of organisms based on short-tag sequences [52]. Jellyfish was used to calculate K-mer depth distribution [53], which depends on the characteristic of the genome, and follows the Poisson distribution. Genome size was calculated using the following empirical formula: *G* = *N* × (*L* − *K* + 1)/(*L* × *M*), where *N* is the number of K-mers, *L* is the reads length, *K* stands for the length of K-mer and *M* stands for the observed peak of K-mer depth. All paired-end reads were used for this analysis with K-mer length of 17 bp, 19 bp, 25 bp, and 31 bp.

All the reads were aligned to the genome by using BWA with default parameters to estimate the level of heterozygosity [54]. For reads with multiple mapping positions, only the best hit was retained. SNPs and Indels were called based on the results of alignment using SAMtools [55]. SNPs and Indels with Phred-scaled quality lower than 10 were filtered.

### 4.3. Genome Assembly and Validating the Assembly

A *de novo* assembly procedure was performed on the clean reads to construct contigs by using SOAPdenovo2 software with the following parameters: the k values in K-mer were set as 31, 43, 51, 53, 57, 63, and 83, considering the unsolved repeats by reads and fill gaps in the scaffolds [56]. The best assemblies with K-mer of 53 were selected according to the scaffold N50 and total length of the assembly. Nextly, we improved the genome assembly with the transcriptome unigenes by using L_RNA_scaffolder [57].

The completeness and accuracy of the genome assembly were evaluated by remapping high quality PE reads to the scaffolds by using bowtie 1.2.0 with parameters of “--rdg 3,1 --rfg 3,1 --gbar 2” [58]. The completeness of the assembly was also examined by mapping unigenes from transcriptomes using BLASTn [59]. The physical coverage of each gene was calculated with the help of SOLAR [60]. The transcriptome data of *E. carinicauda* were collected from the NCBI SRA database (Accession No. SRR1105776). The data were *de novo* assembled into contigs using Trinity (http://trinityrnaseq.sourceforge.net/) [61], and the isoforms were removed by TGICL [62]. The assembled genome was further validated by checking the coverage of the 2675 conserved core genes of Arthropoda. All these core genes were aligned to the assembled genomes using BUSCO program (version v3) [63].

### 4.4. Phylogenetic Analysis

All the Illumina PE transcriptome data of nine decapods, namely, *L. vannamei*, *F. chinensis*, *P. monodon*, *Pandalus latirostris*, *M. rosenbergii*, *N. denticulata*, *Astacus leptodactylus*, *E. sinensis*, and *Portunus trituberculatus* were collected from the SRA database of NCBI (Accession numbers: SRR1039534, SRR653437, SRR346404, SRR1460493-SRR1460495, SRR1460504, SRR1460505, SRR388222, SRR388207, SRR388221, SRR345609-SRR345611, DRR001118-DRR001121, SRR650486, SRR629687, SRR1555734, SRR1576649, SRR1013694, SRR1013696, SRR2087155, SRR768319, and SRR1185328, respectively). The RNA samples in the aforementioned research were extracted from different development stages and various tissue samples, such as hepatopancreas, ovary, muscle, testis, heart, gonad, gills, and pleopod. The transcriptome data were assembled *de novo* into contigs using Trinity, and the isoforms were removed by TGICL. All the unigenes were blasted against the Nr database and conjoined with SOLAR. The sequence of unigenes was cut according to the SOLAR results and translated into amino acid sequences to remove pseudogenes. Amino acid sequences with stop codon involved were removed. The full protein-coding genes of *P. hawaiensis* and *D. pulex* were obtained from NCBI (ftp.ncbi.nlm.nih.gov).

After collecting all the unigenes and protein-coding genes of these species, TreeFam method was used for clustering orthologous gene families [64]. Pair-wise BLASTp alignment was initially used to align all-to-all with an *E*-value cutoff of 1 × 10^−10^. Hcluster_sg was then employed to construct gene families [65]. Among these gene families, single-copy genes were chosen for phylogenetic analysis. ML and BI methods were used for phylogenetic tree construction. For ML-tree construction, sequence alignment was performed using MUSCLE 3.6 [66]. ML analysis was performed on PhyML with the substitution model JTT + gamma + Inv [67]. 1000 bootstraps were conducted to produce the branch support values [24,68]. Mrbayes 3.2.1 was used for BI analysis [69], two independent runs, each with four chains, were analyzed for millions of generations until the standard deviation of split frequencies converged towards zero. The first 25% of the sampled trees were discarded as burn-in.

### 4.5. Allelic SNPs Analysis

To identify whether *E. carinicauda* underwent genome duplication, the analysis of bi-allelic SNPs was carried out according to previous researches [70]. After collecting all the SNPs, only bi-allelic variation was used, which represent for the reference and derived alleles. The alleles frequency was calculated for each bi-allelic SNPs. In a diploid, a unimodal distribution around 50% would be expected. While a trimodal distribution with peaks at 25%, 50% and 75% would be found in a tetraploid.

### 4.6. Analysis of Hox Gene Cluster

The Hox gene cluster of various species contains 10 conserved Hox genes. The sequences of 10 Hox genes of *L. vannamei* and *P. hawaiensis* were downloaded from NCBI. BLASTx program was used to compare *E. carinicauda* unigenes against these Hox genes. The matched unigenes were blasted against Nr database to identify Hox gene sequences. The copy number of these Hox genes was counted when compared with the assembled genome.

### 4.7. Identification of HTGs and NUMTs

BLASTx-based HGT search and phylogenetic analysis were performed on the unigenes of *E. carinicauda* by using the method for identifying HTGs in shrimps [71]. Candidate HTGs were collected from unigenes and blasted against the genome to identify the presence of HTGs.

The mitochondrial genomes of *E. carinicauda* were retrieved from the NCBI database (NC_012566.1) [72]. BLASTn search was performed on the genome against the corresponding mitochondrial genome sequences with the *E* value cutoff of 1 × 10^−^^5^ and match length cutoff of 100 bp. The total number and locations of NUMTs were determined from the BLASTn results. Mitochondrial genome sequences in the assembled genome were determined by the BLASTn results with identity value larger than 98%.

### 4.8. Repeats Annotation and Divergence Time Analysis

RepeatModeler (http://www.repeatmasker.org/RepeatModeler.html) was used for de novo identification of the repeat family and construction of a local repeat database. RepeatMasker was used to identify the TEs by aligning the genome sequences against RepBase (RepBase21.04) and the local library with default parameters [73]. TEs in the genome were determined using a combination of *de novo* based and homology based approaches. Substitution rate of TEs was calculated by RepeatMasker through comparing the genomic and repeat consensus sequences.

## 5. Conclusions

This study provides a valuable genome resource of *E. carinicauda* for research on decapod shrimps, the 5.57 Gb sequences covering about 97% of the genome and more than 95% of coding regions. Full structures of 40,002 unigenes were covered by single scaffold, which benefit for experimental research of economical shrimps, especially for genome editing experiments. We have identified three candidate bacteria-originated HTGs, and 177 NUMTs (totally 67,764 bp) on the genome, which was significantly more than that of *N. denticulata* (35 NUMTs with total length of 4718 bp). Most of these NUMTs originated from mitochondrial genome separately rather than duplication. *E. carinicauda* share a relatively large genome size with many other caridean shrimps, which was larger than many other decapods. Decoding the *E. carinicauda* genome explained the genome size evolution of decapod shrimps. In contrast to transposons, retrotransposons proliferation is responsible for genome size evolution, and furthermore, these retrotransposons appeared to be relatively recent transposed after the divergence of *E. carinicauda* or its ancestor Palaemonidae. RTE-BovB, Jockey, Gypsy, and DIRS are four specifically expanded retrotransposons in the genome. Considering its economic value and experimental manipulability, we believe the genome resources of this species could serve an initial platform for breeding high quality shrimps.

## Figures and Tables

**Table 1:** Genome assembly statistics of *E. carinicauda*.

Criteria	Contig	Scaffold
Number	20,407,032	13,897,062
Total length (bp)	4,865,350,937	5,567,872,237
Longest (bp)	13,513	553,834
Shortest (bp)	100	100
N50 (bp)	263	816
N90 (bp)	116	129
>2 kb	28,741	286,753

**Table 2:** Coverage of unigenes in *E. carinicauda* genome *.

Criteria	Unigenes
Unigene num	81,135
Match unigene num	77,374
Match unigene num (%)	95.36%
90% in one scaf	40,002
90% in one scaf (%)	49.30%
50% in one scaf	68,128
50% in one scaf (%)	83.96%

* “Match unigene num” indicates the number of matched unigenes in blast results against the genome with *E* value cutoff of 1 × 10^−10^. “90% in one scaf” indicates the number of unigenes with 90% of length covered by a single scaffold. “50% in one scaf” indicates the number of unigenes with 50% of length covered by a single scaffold.

**Table 3:** Comparative results of repeats of four crustaceans.

Repeats	*E. carinicauda*	*N. denticulata*	*P. hawaiensis*	*D. pulex*
Total length	5.57 Gb	1.72 Gb	4.02 Gb	197 Mb
GC level	37.47%	35.11%	40.84%	40.77%
Bases masked	1.99 Gb	3.79 Gb	1.49 Gb	40 Mb
Repeat percent	36.37%	22.03%	37.17%	20.45%
SINEs:	0.01%	0.51%	0.03%	0.98%
LINEs:	8.86%	5.07%	6.43%	0.90%
RTE-BovB	3.36%	0.63%	0.19%	0.24%
Jockey	2.12%	0.20%	0.15%	0.05%
L3/CR1	1.65%	2.31%	3.31%	0.00%
LTR elements	5.41%	0.26%	0.58%	5.48%
Gypsy	2.41%	0.21%	0.00%	2.77%
DIRS	2.87%	0.00%	0.00%	0.28%
DNA elements	0.90%	1.14%	4.49%	1.75%
Charlie	0.02%	0.10%	0.13%	0.00%
Tigger	0.48%	0.33%	0.05%	0.02%
Unclassified	19.28%	9.54%	24.31%	10.22%
Total TEs	34.47%	16.52%	35.84%	19.33%
Satellites	0.01%	0.09%	0.04%	0.00%
Simple repeats	1.39%	3.47%	1.27%	0.44%
Low complexity	0.64%	2.00%	0.13%	0.67%

## Supplementary Materials

The following are available online at www.mdpi.com/1660-3397/15/7/213/s1. Table S1. Genome sequencing data of *E. carinicauda*; Table S2. Transcriptome assembly of *E. carinicauda*; Table S3. Transposable elements of *E. carinicauda*. Figure S1. The sequencing quality score distribution along reads. Ec01_L1 stand for the 170 bp library, and Ec01_L2 stand for 500 bp library; Figure S2. The identity distribution between NUMTs and mitochondrial genome. The orange bar indicates the identity distribution of NUMTs against mitochondrial genome; the green bar indicates the identity distribution between different NUMTs; Figure S3. Sequencing depth distribution of the contigs with Hox genes and single-copy genes located. The bar plot indicates the sequencing depth distribution of all contigs.
