# Supplementary material for: Genome Sequences of Marine Shrimp Exopalaemon carinicauda Holthuis Provide Insights into Genome Size Evolution of Caridea
Source: Mar Drugs. 2017 Jul 5;15(7):213. doi: 10.3390/md15070213 (PMC5532655; doi:10.3390/md15070213)

Genome survey of marine shrimp *Exopalaemon carinicauda* Holthuis provide insights into genome size evolution of Caridea

Jianbo Yuan[1,2], Yi Gao[1,2], Xiaojun Zhang[1,2,*], Jiankai Wei[3], Chengzhang Liu[1,2], Fuhua Li[1,2] and Jianhai Xiang [1,2,*]

1. Key Laboratory of Experimental Marine Biology, Institute of Oceanology, Chinese Academy of Sciences, Qingdao 266071, China

2. Laboratory for Marine Biology and Biotechnology, Qingdao National Laboratory for Marine Science and Technology, Qingdao 266071, China

3. Ocean University of China, Qingdao 266071, China

*To whom correspondence should be addressed:

Prof. Jianhai Xiang

Institute of Oceanology, Chinese Academy of Sciences

7, Nanhai Road, Qingdao 266071, China

Tel: + 86-532-82898568

Fax: + 86-532-82898578

E-mail: jhxiang@qdio.ac.cn

Dr. Xiaojun Zhang

Institute of Oceanology, Chinese Academy of Sciences

7, Nanhai Road, Qingdao 266071, China

Tel: + 86-532-82898570

E-mail: xjzhang@qdio.ac.cn

Table S1 Genome sequencing data of *E. carinicauda*

Name	Insert	Raw_Bases(bp)	Clean_Base(bp)	Q20	Q30	GC_Content
Ec01_L1	170	45,870,832,400	43,581,088,000	98.04%	94.58%	39.00%
Ec01_L2	500	47,504,188,000	44,562,170,000	97.75%	93.43%	41.26%

Table S2 Transcriptome assembly of *E. carinicauda*

Unigene number:	81,135
Total length:	86,343,999
Longest:	27,586
Shortest:	301
N50:	1,712
N90:	417
>2kb:	10,838
<=500bp:	33,715

Table S3 Transposable elements of *E. carinicauda*

total length: 5,567,872,237 bp				
GC level: 37.47 %				
bases masked: 1,996,943,900 bp (36.37 %)				
		**Number**	**Length**	**Percentage**
SINEs:		6195	681246	0.01%
LINEs:		2919742	486442140	8.86%
	LINE2	143287	23158588	0.42%
	L3/CR1	519864	90529032	1.65%
	RTE-BovB	1059487	184498893	3.36%
	Jockey	668244	116367901	2.12%
LTR elements:		1811582	297116723	5.41%
	ERV_classI	5307	852766	0.02%
	ERV_classII	6296	1297125	0.02%
	Gypsy	806323	132244661	2.41%
	DIRS	960927	157601191	2.87%
DNA elements:		353988	49419683	0.90%
	hAT-Charlie	9330	1331247	0.02%
	TcMar-Tigger	182921	26447722	0.48%
Unclassified:		9908762	1058444863	19.28%
**Total interspersed repeats:**			1892104655	34.47%
Small RNA:		9451	807660	0.01%
Satellites:		2510	460053	0.01%
Simple repeats:		1637540	76153607	1.39%
Low complexity:		695902	35056642	0.64%

Figure S1. The sequencing quality score distribution along reads. Ec01_L1 stand for the 170 bp library, and Ec01_L2 stand for 500 bp library.

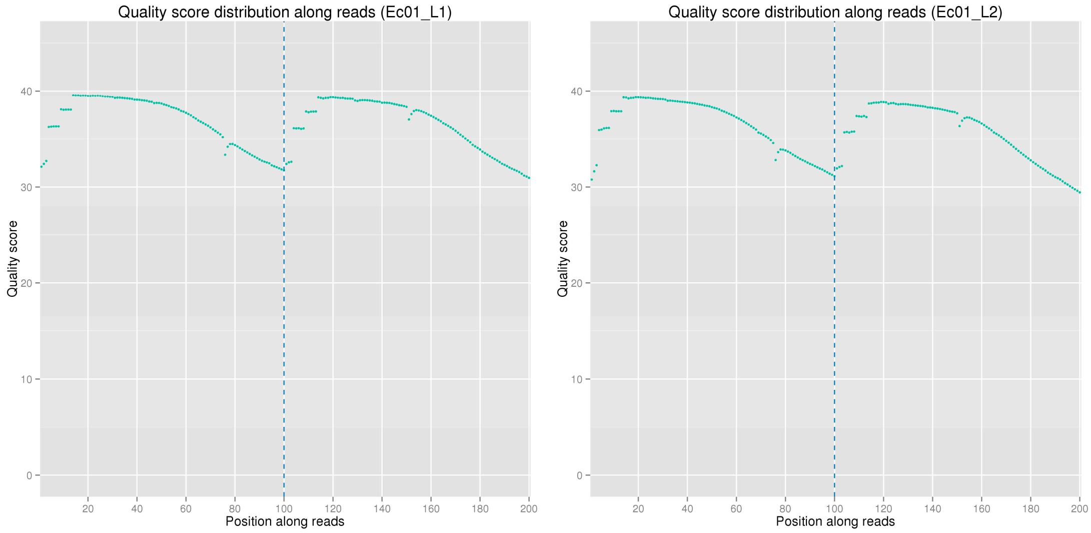

Figure S2. The identity distribution between NUMTs and mitochondrial genome. The orange bar indicates the identity distribution of NUMTs against mitochondrial genome; the green bar indicates the identity distribution between different NUMTs.

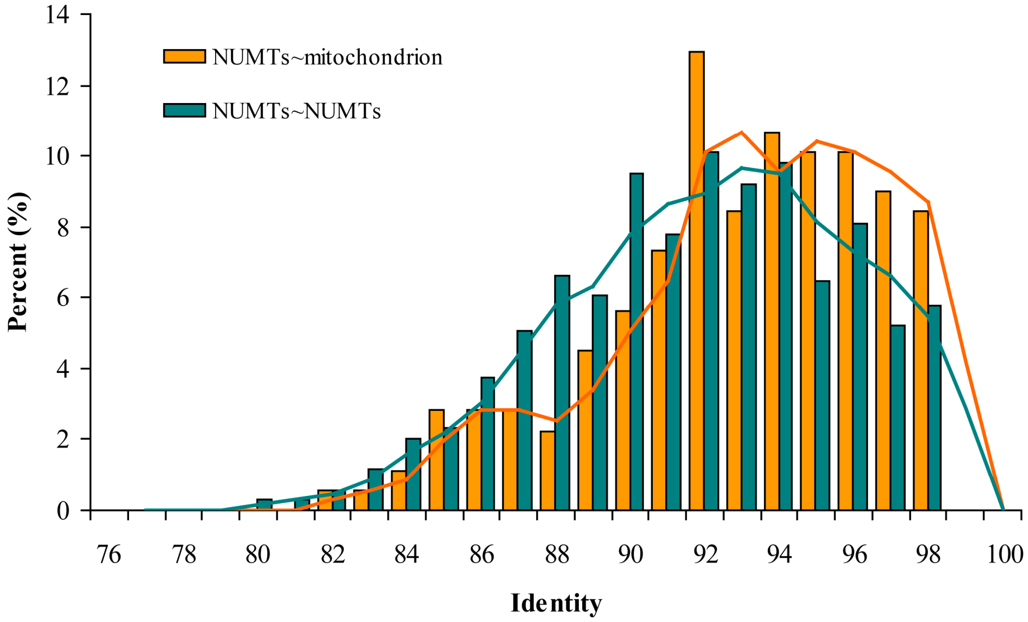

Figure S3. Sequencing depth distribution of the contigs with Hox genes and single-copy genes located. The bar plot indicates the sequencing depth distribution of all contigs.

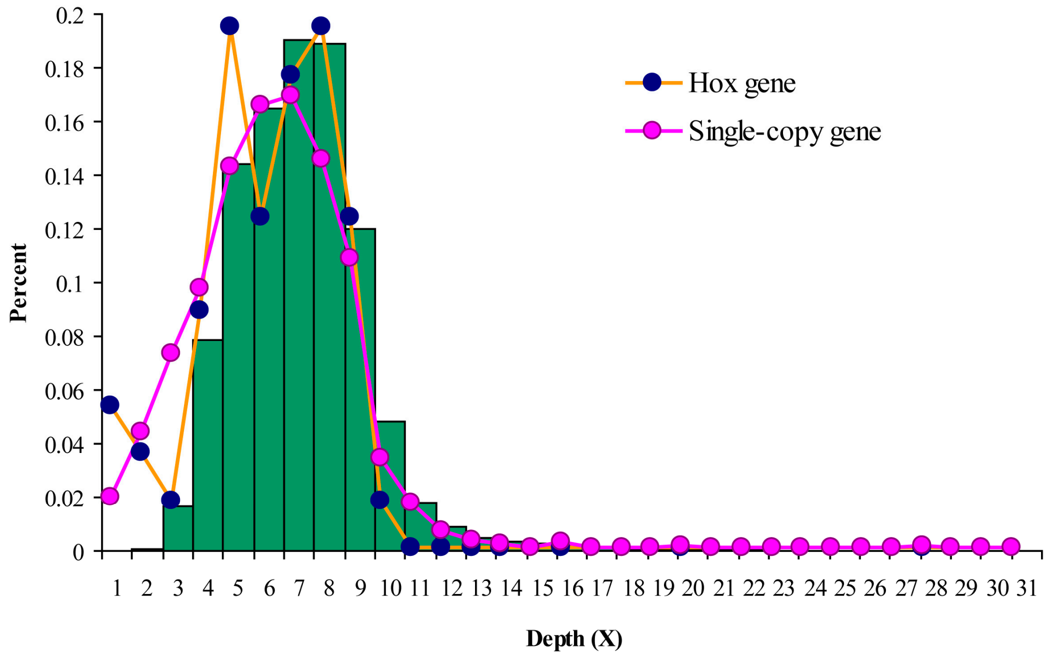

Supplement: Supplementary file 1 [file marinedrugs-15-00213-s001.pdf]
